# Genome-Wide Characterization and Expression Profiling of MADS-Box Family Genes Under Abiotic Stress in *Arundo donax*

**DOI:** 10.3390/plants15213267

**Published:** 2026-10-23

**Authors:** Mengmeng Ren, Zeqing Xin, Yuxin Zhuang, Shaopeng Zhang

**Affiliations:** National R&D Center for Se-Rich Agricultural Products Processing, School of Modern Industry for Selenium Science and Engineering, Wuhan Polytechnic University, Wuhan 430048, China; ren_mm1991@163.com (M.R.); 15688609338@163.com (Z.X.); 13316030370@163.com (Y.Z.)

**Keywords:** *Arundo donax*, MADS-box, gene family, abiotic stress, expression pattern

## Abstract

MADS-box transcription factors play crucial roles in plant growth, development, and response to abiotic stresses. However, their functions in abiotic stress resistance remain largely unexplored in the bioenergy crop *Arundo donax* (*A. donax*). In this study, we identified and comprehensively analyzed 97 MADS-box genes (*AdMADS*) in the *A. donax* genome. Phylogenetic analysis classified these genes into type I (44 members) and type II (53 members) subfamilies, which were further subdivided into 12 subgroups. Comparative analysis revealed clear distinctions between type I and type II genes in terms of the physicochemical properties of their encoded proteins and their exon-intron structures. Promoter analysis identified numerous cis-regulatory elements associated with light responsiveness, plant hormones, and stress signals. Gene duplication analysis suggested that whole-genome duplication/segmental duplication was the major detected contributor to the expansion of the *AdMADS* gene family, with type II genes contributing predominantly to these duplication events. Collinearity analysis demonstrated a high conservation of MADS-box genes among *A. donax*, rice, and foxtail millet, reflecting their evolutionary conservation within Gramineae. By analyzing public RNA-seq data, we identified 45 *AdMADS* genes as stress-responsive candidates under waterlogging, drought, and salt stress, and selected candidates were further validated by RT-qPCR. Moreover, AdMADS33 and AdMADS53 were found to bind to and activate the promoter of the ABA synthesis gene *AdNCED* in tobacco, suggesting that they may contribute to salt tolerance by promoting *AdNCED* transcription. This study provides a valuable resource for future functional gene mining and offers a solid foundation for the molecular breeding of *A. donax*.

## 1. Introduction

*Arundo donax* L., commonly known as giant reed, is a perennial rhizomatous grass that has garnered increasing global interest for its exceptional biomass productivity and resilience in marginal lands [1,2,3]. It exhibits a rapid growth rate, high biomass yield, and broad adaptability to diverse environmental conditions, including drought, salinity, and heavy metal stresses [4,5]. These attributes establish it as a prime candidate for bioenergy production and phytoremediation. However, despite its agronomic and economic potential, the molecular mechanisms underlying its key traits, such as biomass composition and stress tolerance, remain largely unexplored.

The MADS-box gene family encodes a large group of transcription factors (TFs) that play pivotal roles in diverse developmental processes across eukaryotes, including plants, animals, and fungi [6]. This family is named for the initial letters of its first four identified members: MCM1 in *Saccharomyces cerevisiae*, AGAMOUS in *Arabidopsis thaliana* (*A. thaliana*), DEFICIENS in *Antirrhinum majus*, and SRF in *Homo sapiens* [7,8]. A defining feature of these TFs is a highly conserved DNA-binding domain of approximately 60 amino acids, known as the MADS-domain. This domain binds to specific CArG-box sequences [C-(A/T) rich-G-G] to regulate downstream gene expression [9,10]. In plants, MADS-box genes are divided into two major types based on phylogenetic and structural characteristics. Type I genes (M-type) have a simpler structure, typically containing only a MADS domain, and are subdivided into Mα, Mβ, and Mγ subgroups [11]. Type II genes (MIKC-type), however, exhibit a modular structure comprising four domains: the MADS (M) domain, an Intervening (I) domain, a Keratin-like (K) domain that facilitates protein dimerization, and a highly variable C-terminal (C) domain [12]. These Type II genes are further subdivided into MIKC^C^ and MIKC* subgroups [13].

MADS-box transcription factors are well-established master regulators of plant growth and development, controlling flowering, fruit development, and organ identity as described in the ABCDE model [14]. Beyond development, they also integrate environmental signals into stress response pathways. In Arabidopsis, AGL16 negatively regulates salt tolerance by repressing heat shock protein synthesis and ABA signaling, whereas its rice ortholog OsMADS27 enhances salt tolerance by activating *OsHKT1;1* and *OsSPL7* [15,16]. Besides, OsMADS23 directly upregulates ABA and proline biosynthesis genes, such as *OsNCED2* and *OsP5CR*, to confer drought and salt tolerance [17], and OsMADS25 improves salt and oxidative stress tolerance by modulating ABA signaling and ROS scavenging [18]. MADS-box genes also participate in cold stress responses. In pepper, CaMADS positively regulates cold tolerance [19], and in apple, MdDAM1 promotes cold tolerance and growth cessation [20]. Collectively, these findings highlight the diverse roles of MADS-box transcription factors in abiotic stress responses. Although such roles have been documented in various species, MADS-box genes remain systematically unstudied in the promising bioenergy crop *A. donax*.

In this study, we performed the first genome-wide survey to identify all putative MADS-box genes in *A. donax*, followed by a comprehensive characterization of their chromosomal locations, phylogenetic relationships, gene structures, conserved domains and motifs, and duplication events. Furthermore, we investigated their expression patterns in response to various abiotic stresses and revealed that AdMADS33 and AdMADS53 may contribute to salt tolerance by activating the ABA synthesis gene *AdNCED*. Our findings lay a solid foundation for the future functional characterization of MADS-box genes in *A. donax* and provide crucial insights into their roles in mediating stress tolerance.

## 2. Result

### 2.1. Identification, Properties, and Chromosomal Locations of the AdMADS Genes

**Figure 1:**
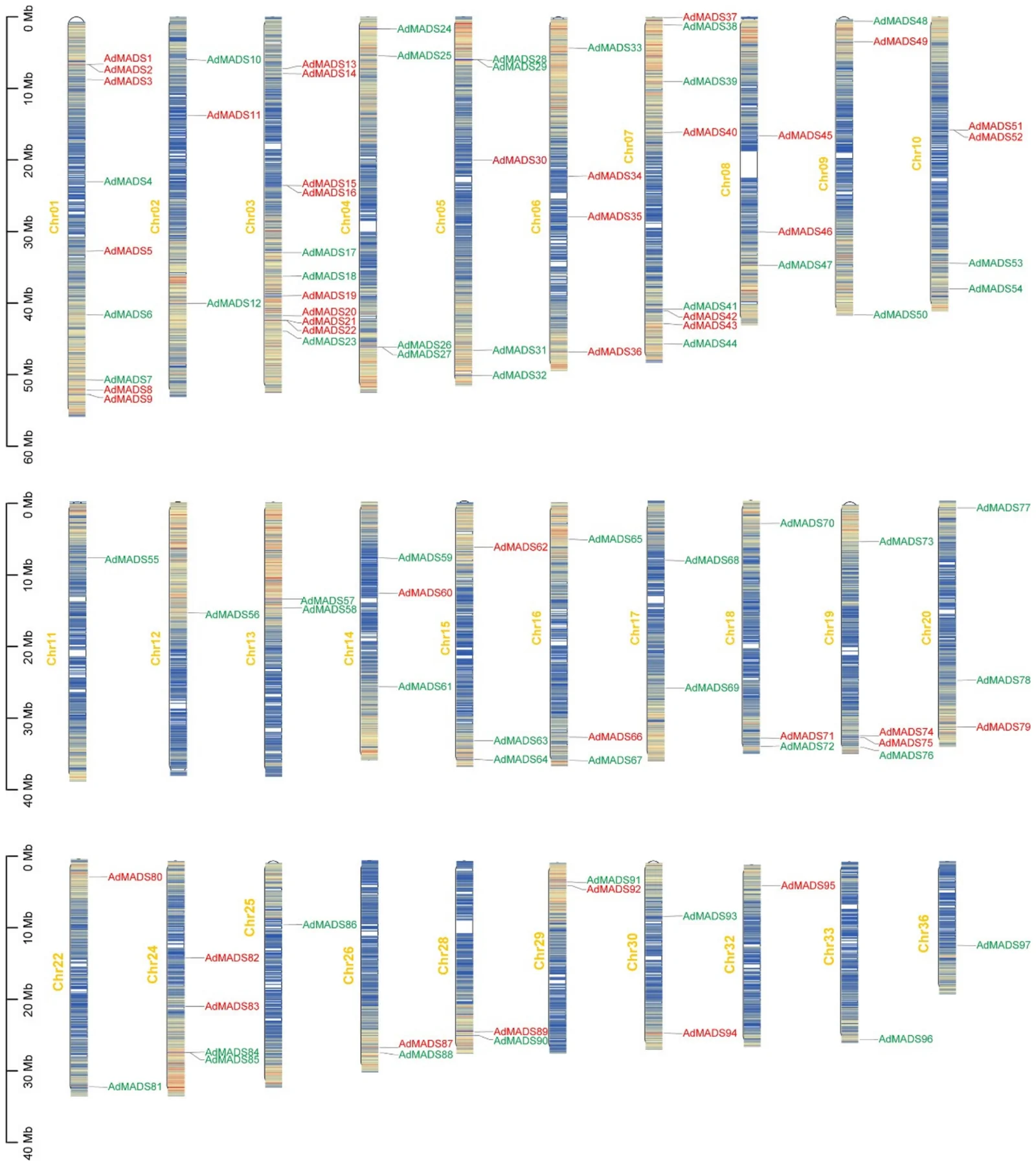
Chromosomal locations of the 97 *AdMADS* genes on the *A. donax* genomes. Type I and type II *AdMADS* genes are marked with red and green characters. The scales on the left denote the chromosome positions. Different colored bands in chromosomes represent gene distribution densities.

Combining BLAST v2.16.0 and HMM searches v3.4, a total of 97 members of the MADS-box gene family were identified from the *A. donax* genome. These genes are unevenly distributed on 30 chromosomes, with chromosomes 3 (11), 1 (9), and 7 (8) having the most *AdMADS* gene members, while no MADS gene is distributed on chromosomes 21, 23, 27, 31, 34, and 35 (Figure 1). According to the domain identification with Pfam, the 97 AdMADS proteins were grouped into 44 type I (MADS-box domain) and 53 type II (MADS-box and K-box domain) subfamilies. The amino acid numbers of AdMADS proteins range from 65 to 633, with predicted molecular weights ranging from 15,537 to 71,214 Da. The theoretical isoelectric points range from 4.68 to 9.85, and instability ranges from 32.9 to 77.66. All AdMADS proteins are hydrophilic, with a grand average hydropathicity ranging from −7.008 to −0.006 (Table S2). Interestingly, there are significant differences between the two types of AdMADS proteins. Type I AdMADS proteins overall have a higher number of amino acids (300.9/238.4), higher molecular weight (32,682.7/27,299.6 Da), lower isoelectric point (7.000/7.979), slightly lower instability (53.94/54.72), and higher hydropathicity (−0.3873/−0.6372) than those of type II AdMADS proteins.

Combining BLAST and HMM searches, a total of 97 members of the MADS-box gene family were identified from the *A. donax* genome. These genes were unevenly distributed across 30 chromosomes, with chromosomes 3 (11 genes), 1 (9 genes), and 7 (8 genes) harboring the most *AdMADS* members, while no genes were located on chromosomes 21, 23, 27, 31, 34, and 35 (Figure 1). Based on domain identification using Pfam, the 97 AdMADS proteins were classified into 44 type I (containing only the MADS-box domain) and 53 type II (containing both MADS-box and K-box domains) subfamilies. The number of amino acids in AdMADS proteins ranged from 65 to 633, with predicted molecular weights ranging from 15.5 to 71.2 kDa. The theoretical isoelectric points (PI) ranged from 4.68 to 9.85, and the instability indices ranged from 32.9 to 77.66. All AdMADS proteins were hydrophilic, with grand averages of hydropathicity (GRAVY) ranging from −0.637 to −0.006 (Table S2). Interestingly, significant differences were observed between the two types of AdMADS proteins. Compared to type II proteins, type I AdMADS proteins generally contained more amino acids, had higher molecular weights, lower theoretical PI, and a slightly lower instability index. Moreover, type I proteins showed a less negative grand average of hydropathicity, indicating relatively lower hydrophilicity (Table 1).

### 2.2. Phylogenetic Relationship of the AdMADS Proteins

**Figure 2:**
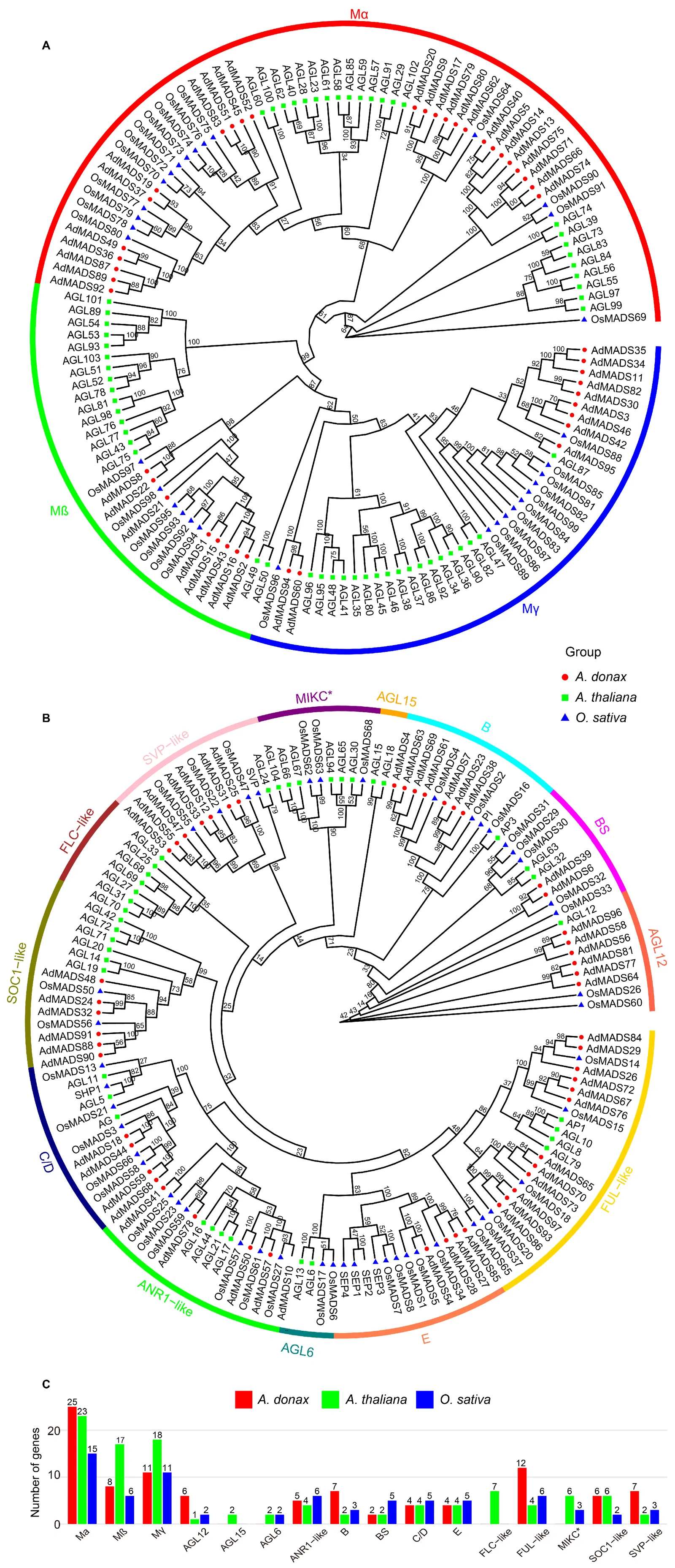
Phylogenetic relationships of the MADS-box proteins. Phylogenetic trees of MADS-box proteins from *A. donax*, *O. sativa*, and *A. thaliana* for Type I (**A**) and Type II (**B**) groups, constructed using the maximum-likelihood method with IQ-TREE. The corresponding proteins in *A. donax, O. sativa,* and *A. thaliana* are marked with red dots, green squares, and blue triangles, respectively. Different subgroups are indicated by stripes with different colors. (**C**) Distribution of MADS-box gene subgroups in *A. donax* (red), *A. thaliana* (green), and *O. sativa* (blue). The number of genes belonging to specific subgroups is indicated above each column.

To elucidate the phylogenetic relationships, we constructed separate phylogenetic trees for type I and type II MADS-box proteins from *A. donax*, *Oryza sativa* (*O. zativa*), and *A. thaliana*. The type I AdMADS proteins were classified into three subgroups: Mα (25 members), Mβ (8 members), and Mγ (11 members) (Figure 2A). Similarly, the type II proteins were resolved into nine distinct subgroups (Figure 2B): ALG12 (6), ANR1-like (5), B (7), BS (2), C/D (4), E (4), FUL-like (12), SOC1-like (6), and SVP-like (7). Notably, *A. donax* and *O. zativa* shared a highly similar distribution pattern of MADS-box proteins across these subgroups. Among all subgroups, the *A. donax* genome harbored the largest number of members in the type I Mα and the type II FUL-like subgroups, a pattern also observed in rice. This conserved distribution is consistent with the close evolutionary relationship between *A. donax* and *O. zativa* (Figure 2C).

### 2.3. Gene Structure and Conserved Domains and Motifs of AdMADS Genes

**Figure 3:**
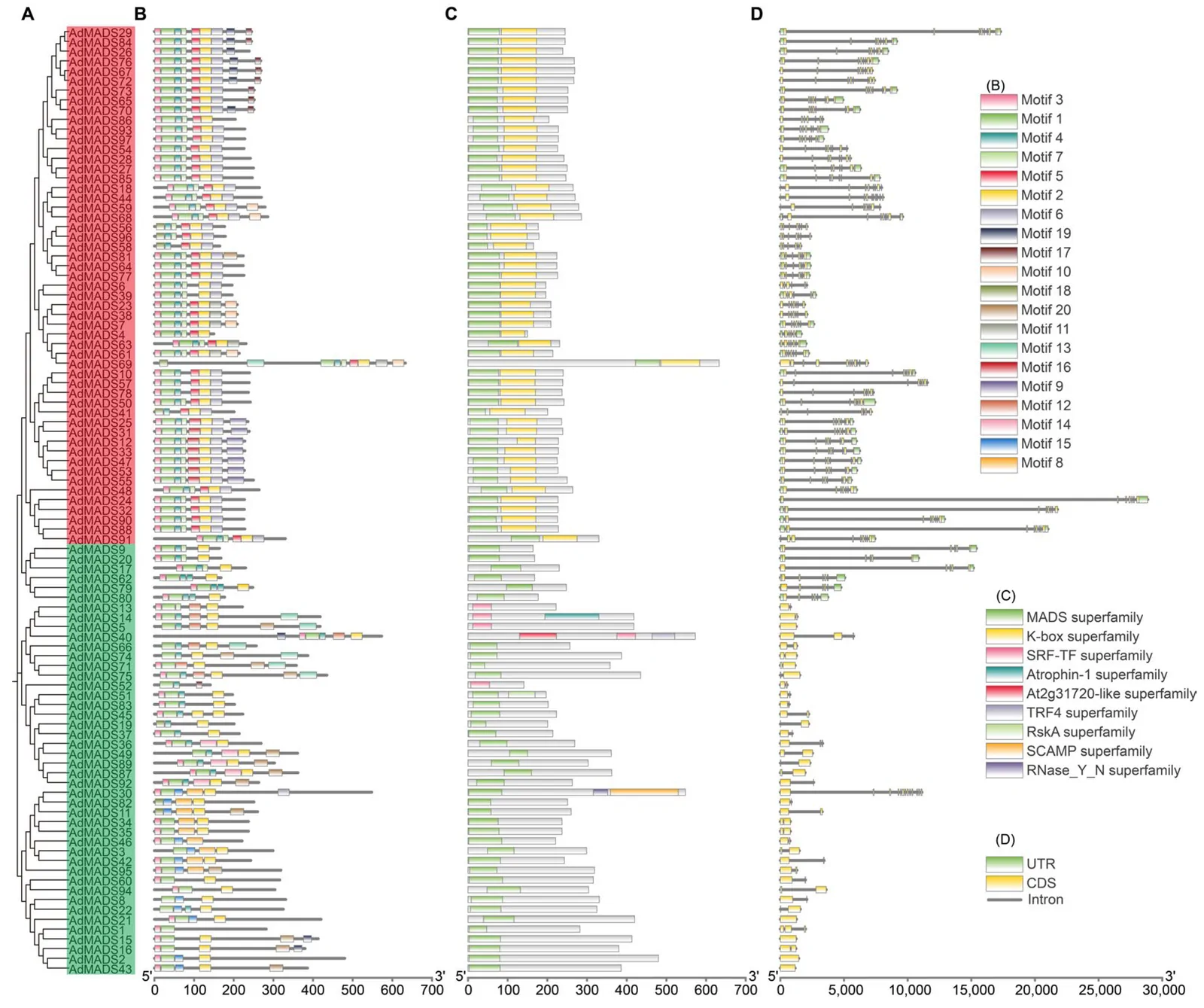
The phylogenetic relationship, domain composition, and gene structure analyses of the *AdMADS* gene family. (**A**) Phylogenetic tree of AdMADS proteins. Type I and Type II groups are highlighted in red and green, respectively. (**B**) Distribution of predicted motifs in AdMADS proteins. The 20 distinct motifs are denoted by different colored boxes. (**C**) Conservation of domains in AdMADS proteins. Different domains are marked by different colored boxes. (**D**) Gene structure of the *AdMADS* gene family. Exons and introns are shown as rectangles and lines, respectively.

To gain comprehensive insight into the sequence features of AdMADS proteins, we conducted a systematic analysis guided by a phylogenetic framework, which clearly distinguished type I and type II clades (Figure 3A). Motif prediction revealed that motifs 1–4 were common core elements, present in 92.8% (90/97), 89.7% (87/97), 77.3% (75/97), and 76.3% (74/97) of all AdMADS proteins, respectively (Figure 3B, Table S3). In contrast, motifs 5, 6, and 7 exhibited a distinct subfamily-specific distribution. They were predominantly found in type II proteins, with frequencies of 77.8% (42/54), 79.6% (43/54), and 87.0% (47/54), respectively. Conversely, none of the type I proteins contained motif 5 or 6, and only 11.6% (5/43) contained motif 7 (Figure 3B, Table S3). Domain analysis corroborated the HMM-based classification, confirming that all AdMADS proteins contain a MADS-box or SRF-TF domain, while type II proteins additionally feature a K-box domain (Figure 3C, Table S3).

Substantial differences in gene structure were also observed. Type I genes exhibited a simpler architecture, with 77.3% (34/43) containing zero or one intron. In contrast, all type II genes possessed five or more exons (Table S3). Although the average exon length was comparable between the two types, the introns of type II genes were, on average, substantially longer than those of type I genes (5898.9 bp vs. 1989.3 bp) (Figure 3D, Table S3).

### 2.4. Cis-Regulator Elements in Promoters of AdMADS Genes

**Figure 4:**
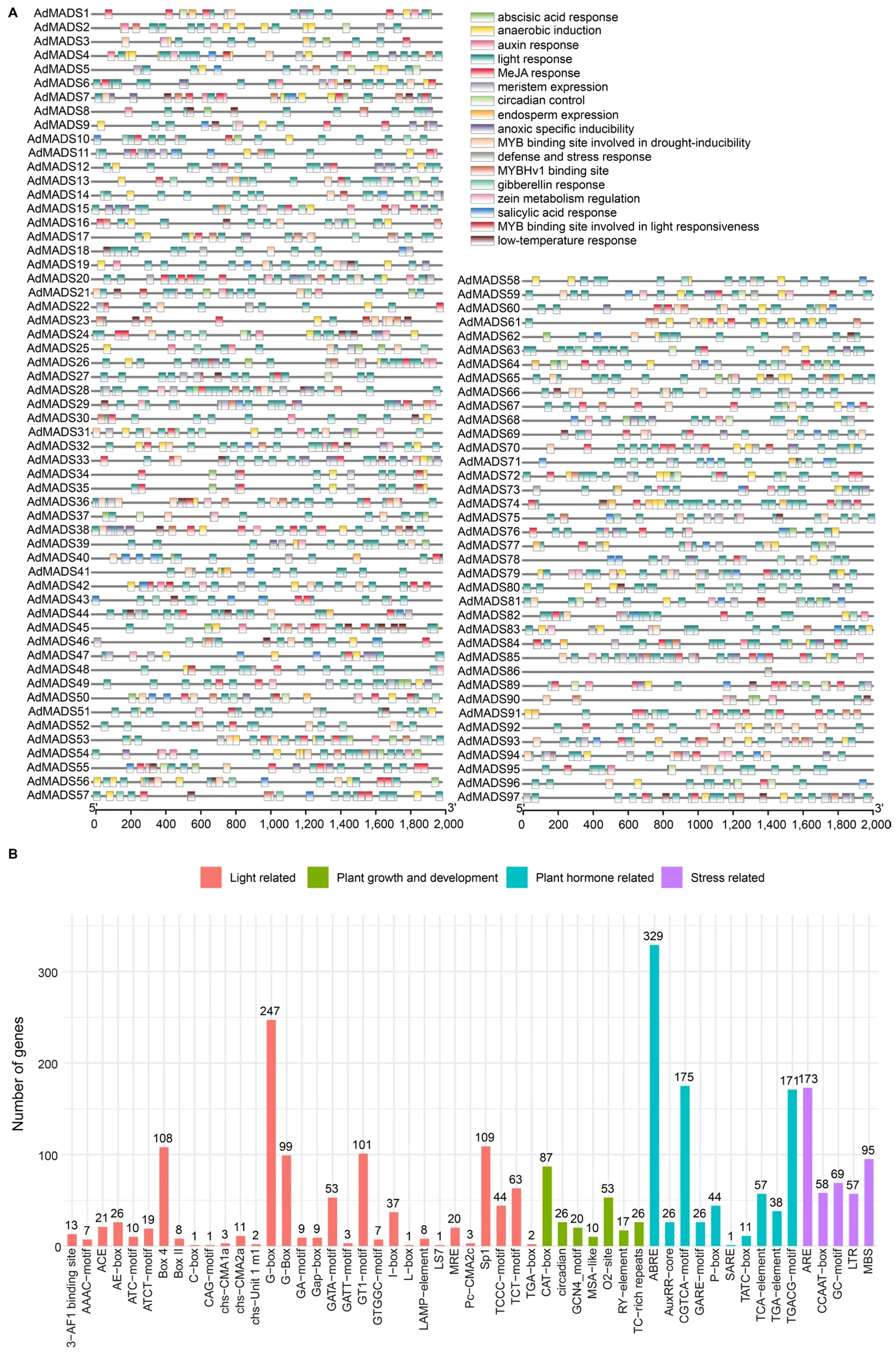
Identification of cis-regulatory elements in the promoters of *AdMADS* genes. (**A**) Distribution of cis-regulatory elements in the *AdMADS* gene promoters. Different elements are represented by different colored boxes. (**B**) Statistical analysis of the identified cis-regulatory elements. The number of genes containing a specific element is indicated above each column. Elements are color-coded according to their predicted function.

To elucidate the transcriptional regulation of *AdMADS* genes, we extracted the promoter sequences and predicted cis-regulatory elements using the PlantCARE database. A total of 50 distinct types of cis-acting elements were identified, with an average of 26.1 elements per gene. Among them, *AdMADS20* (64 elements) contained the highest number of elements, followed by *AdMADS79* (58) and *AdMADS85* (52). In contrast, no element was predicted in the promoters of *AdMADS87* and *AdMADS88* (Figure 4A and Table S4).

These elements were classified into four functional categories: light responsiveness, plant growth and development, hormone response, and stress response. Light-responsive elements were the most abundant category, detected in nearly all *AdMADS* promoters, which may indicate a potential role of light in regulating the MADS-box gene family. For instance, the “G-box” (247 elements) was present in 82.5% (80/97) of the promoters, and “Box 4” (108 elements) was found in 67.0% (65/97). Within the plant hormone category, elements related to methyl jasmonate (MeJA) responsiveness (CGTCA and TGACG motifs) were the most prevalent (346 occurrences), followed by abscisic acid (ABA)-responsive elements (ABRE, 329 occurrences). These findings suggest that MeJA and ABA may potentially be involved in the hormonal regulation of *AdMADS* genes, although this possibility requires further experimental validation. Stress-related elements were also widespread, including 242 associated with anaerobic induction (ARE and GC-motif), 95 with drought inducibility (MBS), and 57 with low-temperature response (LTR) (Figure 4B). Collectively, these results indicate that the expression of *AdMADS* genes is likely regulated by a combination of external environmental and internal physiological signals.

### 2.5. Intraspecific and Interspecific Collinearity Relationship of AdMADS Genes

**Figure 5:**
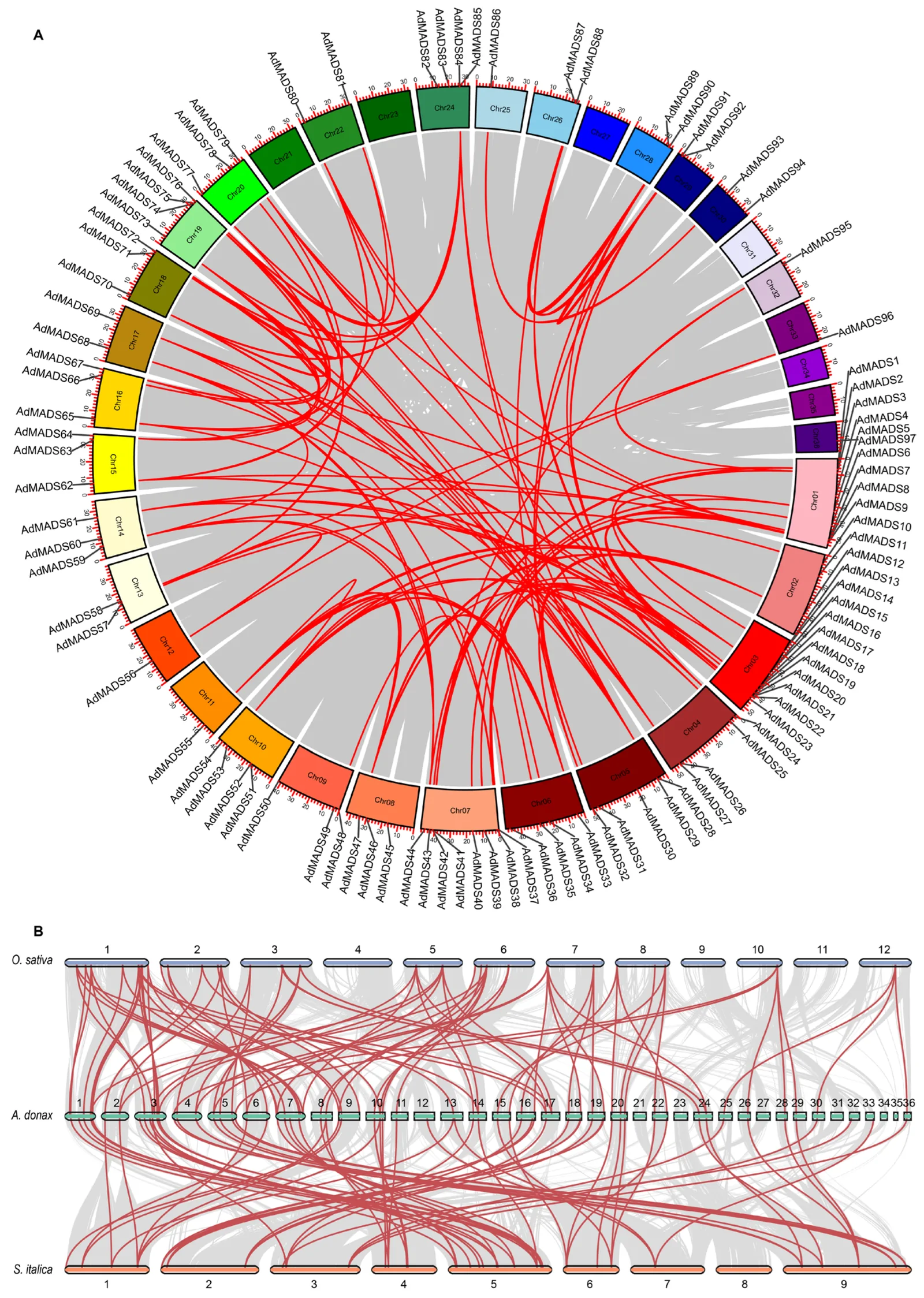
Syntenic analyses of *AdMADS* genes. (**A**) Collinearity relationships of the *AdMADS* genes. Red lines link two syntenic *AdMADS* genes. (**B**) Syntenic relationships of MADS-box genes of *A. donax*, *O. sativa* and *S. italica*. The red lines represent the MADS-box genes, and the grey lines denote other syntenic genes.

Previous studies have indicated that the *A. donax* genome has undergone two whole-genome duplication (WGD) events [21]. To investigate the contribution of WGD to the expansion of the MADS-box gene family, we analyzed the collinearity relationship of the 97 *AdMADS* genes. A total of 97 collinear gene pairs, all predicted to arise from WGD or segmental duplication, were identified among 72 *AdMADS* genes (Figure 5A and Table S5). Among all *AdMADS* genes, *AdMADS28* had the highest number of homologous genes (6), followed by *AdMADS26* and *AdMADS84*, each with five. Notably, all duplicated gene pairs were confined within the same type, indicating that the divergence between type I and type II *AdMADS* genes predated the WGD events in *A. donax*, which is consistent with previous findings [22]. Furthermore, a higher proportion of type II genes (79.2%, 43/54) were involved in duplication events compared to type I genes (67.4%, 29/43), consistent with the reported higher evolutionary conservation of type II MADS-box genes [23]. The Ka/Ks ratios for all duplicated gene pairs were less than 1.0 (Table S5), indicating that *AdMADS* genes have undergone strong purifying selection to maintain critical biological functions. The estimated timing of these duplication events ranged from 28.86 to 0.35 million years ago (Table S5), which is consistent with the proposed timing of the second WGD in *A. donax* [21].

To further investigate the syntenic relationships of MADS-box genes, we compared *AdMADS* with their orthologs in two other gramineous species, *Oryza sativa* and *Setaria italica* (*S. italica*). We identified 81 collinear gene pairs between *A. donax* and *O. sativa*, involving 68 *AdMADS* and 30 *OsMADS* genes; and 79 collinear gene pairs were identified between *A. donax* and *S. italica*, involving 74 *AdMADS* and 38 *SiMADS* genes (Figure 5B and Table S6). A majority of these *OsMADS* (83.3%) and *SiMADS* (65.8%) genes had two or more orthologs in the *A. donax* genome, consistent with the allopolyploid nature of the *A. donax* genome [21].

### 2.6. Identification of AdMADS Genes Related to Abiotic Stress

**Figure 6:**
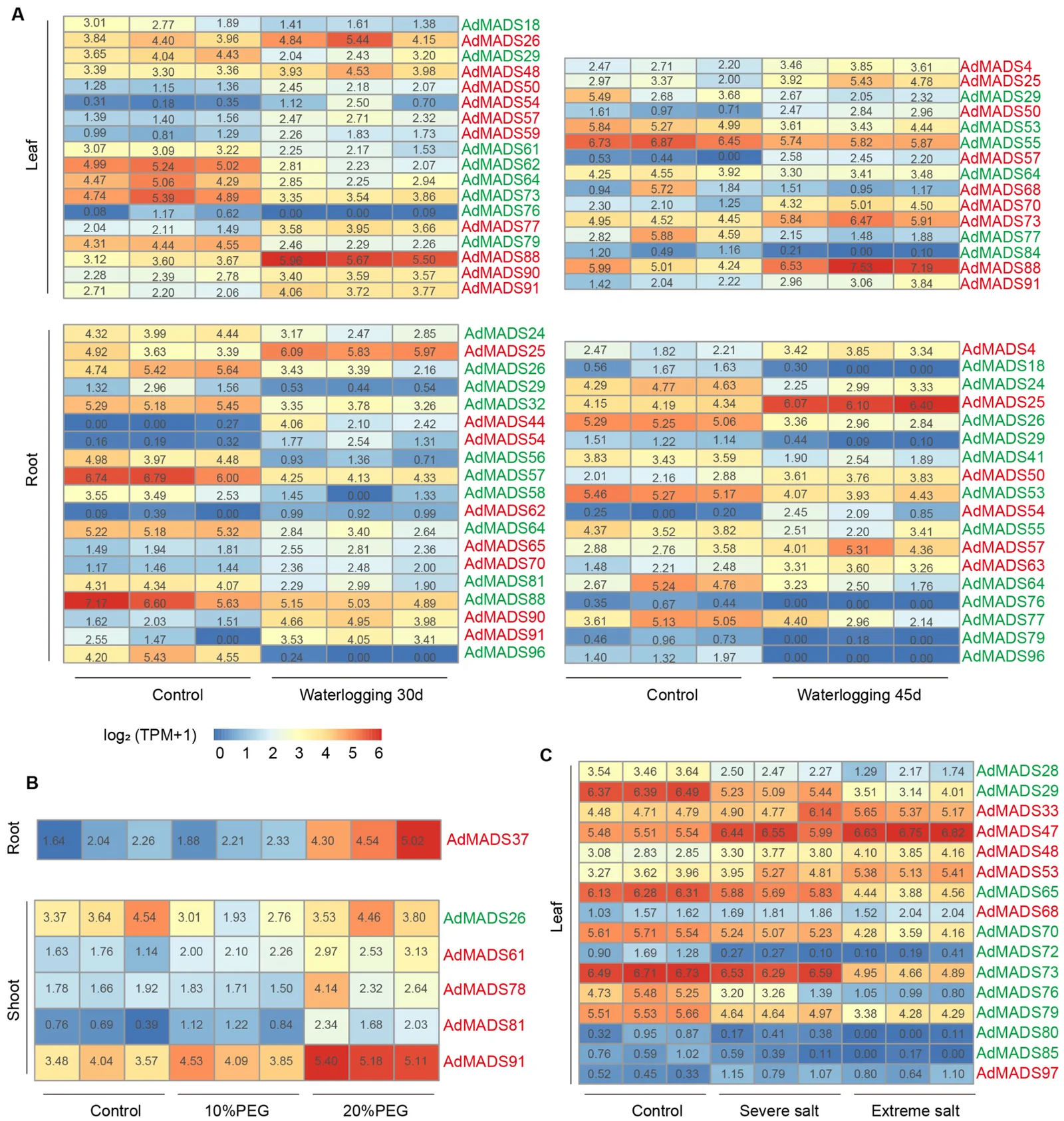
Identification of *AdMADS* genes responsive to abiotic stress. Heatmaps showing differentially expressed *AdMADS* genes under (**A**) waterlogging, (**B**) drought, and (**C**) salt stress. Each cell represents the log2-transformed transcripts per million (TPM) value (Red: high, Blue: low). *n* = 3 biological replicates for RNA-seq experiment. Up-regulated and down-regulated genes are indicated by red and green text, respectively.

Given the importance of abiotic stress tolerance in *A. donax* as a bioenergy crop, we investigated the potential role of the MADS-box gene family by analyzing their expression patterns under waterlogging, drought, and salt stresses using public RNA-seq datasets. A high average mapping rate of 85.93% (range: 69.11–90.42%) was achieved for all datasets, demonstrating the overall quality and appropriateness of the sequencing data for downstream analyses (Table S7). Under waterlogging stress (PRJNA1069878) [24], the number of differentially expressed *AdMADS* genes varied with treatment duration and tissue type. At 30 days, 18 (10 up-regulated and 8 down-regulated) and 19 (8 up-regulated and 11 down-regulated) genes responded in leaves and roots, respectively; by 45 days, these numbers changed to 15 (9 up-regulated and 6 down-regulated) in leaves and 18 (6 up-regulated and 12 down-regulated) in roots. A subset of eight genes (*AdMADS29*, *AdMADS50, AdMADS57*, *AdMADS64*, *AdMADS73*, *AdMADS77*, *AdMADS88*, *AdMADS91*) were consistently regulated in leaves across both time points, while another eight (*AdMADS24*, *AdMADS25*, *AdMADS26*, *AdMADS29*, *AdMADS54*, *AdMADS57*, *AdMADS64*, *AdMADS96*) showed consistent responses in roots, suggesting their roles in both short-term and long-term waterlogging adaptation (Figure 6A and Figure S1A and Table S8). Under drought stress (PRJEB10039) [25], the response was more limited. Mild drought (10% PEG) triggered no changes in roots and only down-regulated *AdMADS26* in shoots. Severe drought (20% PEG) induced the up-regulation of *AdMADS37* in roots and four genes (*AdMADS61*, *AdMADS78*, *AdMADS81*, *AdMADS91*) in shoots (Figure 6B and Table S8). Salt stress (PRJNA497781) [26] elicited a stronger transcriptional response. Under 256.67 mM NaCl, seven genes were differentially expressed in leaves, a number that increased to sixteen under 419.23 mM NaCl. Six genes (*AdMADS28*, *AdMADS29*, *AdMADS47*, *AdMADS53*, *AdMADS72*, *AdMADS76*) were commonly regulated under both salinity levels, indicating their potential core function in salt stress response (Figure 6C and Figure S1B and Table S8). In total, 45 *AdMADS* genes were differentially expressed under at least one stress condition, indicating that MADS-box genes are widely responsive to abiotic stress in *A. donax*.

**Figure 7:**
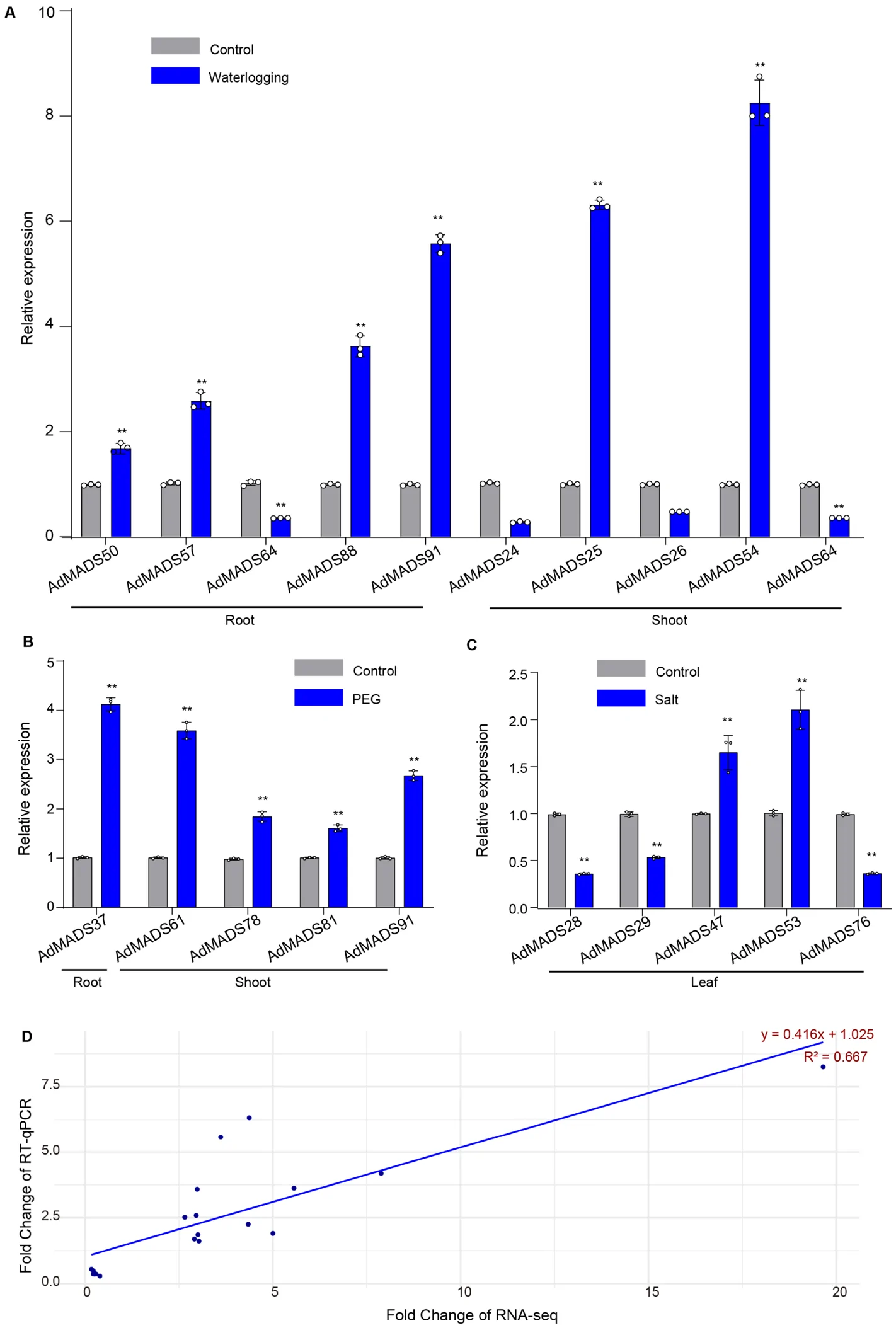
Relative expression of *AdMADS* genes under abiotic stress. (**A**–**C**) Expression levels of selected *AdMADS* genes under (**A**) waterlogging, (**B**) drought (20% PEG), and (**C**) salt (400 mM NaCl) treatments, as determined by RT-qPCR. Data are presented as mean ± SD (*n* = 3 biological replicates). Asterisks indicate a significant difference as determined by Student’s *t*-test (** *p* < 0.01). (**D**) Correlation between RNA-seq and RT-qPCR data. The fold-change values of *AdMADS* gene expression from both methods were subjected to linear regression analysis. The regression equation was calculated in R, and the coefficient of determination (R^2^) was used to measure the fitness of the linear model.

To validate the RNA-seq findings, we quantified the expression levels of selected *AdMADS* genes under abiotic stress conditions using RT-qPCR. The results confirmed stress-specific expression patterns for these candidates. Under waterlogging stress, *AdMADS50*, *AdMADS57*, *AdMADS88*, and *AdMADS91* were up-regulated in roots, while *AdMADS64* was down-regulated. In shoots, *AdMADS24* and *AdMADS54* were up-regulated, whereas *AdMADS26* and *AdMADS64* were down-regulated (Figure 7A). Under drought stress, five *AdMADS* genes (*AdMADS37, AdMADS61*, *AdMADS78*, *AdMADS81*, *AdMADS91*) were up-regulated in either root or shoot tissues (Figure 7B). Under salt stress, *AdMADS47* and *AdMADS53* were up-regulated, while *AdMADS28*, *AdMADS29*, and *AdMADS76* were down-regulated (Figure 7C). To quantitatively assess the consistency between the RNA-seq and RT-qPCR results, we performed a linear regression analysis based on the fold-change values obtained from both methods. The analysis revealed a moderate yet significant positive correlation, described by the equation y = 0.416x + 1.025, with a coefficient of determination (R^2^) of 0.667 (Figure 7D). This significant correlation confirms the reliability of the RNA-seq data and supports its utility for the preliminary screening of candidate genes involved in other abiotic stress responses.

### 2.7. AdMADS33 and AdMADS53 Activate the AdNCED Promoter in Heterologous Systems

**Figure 8:**
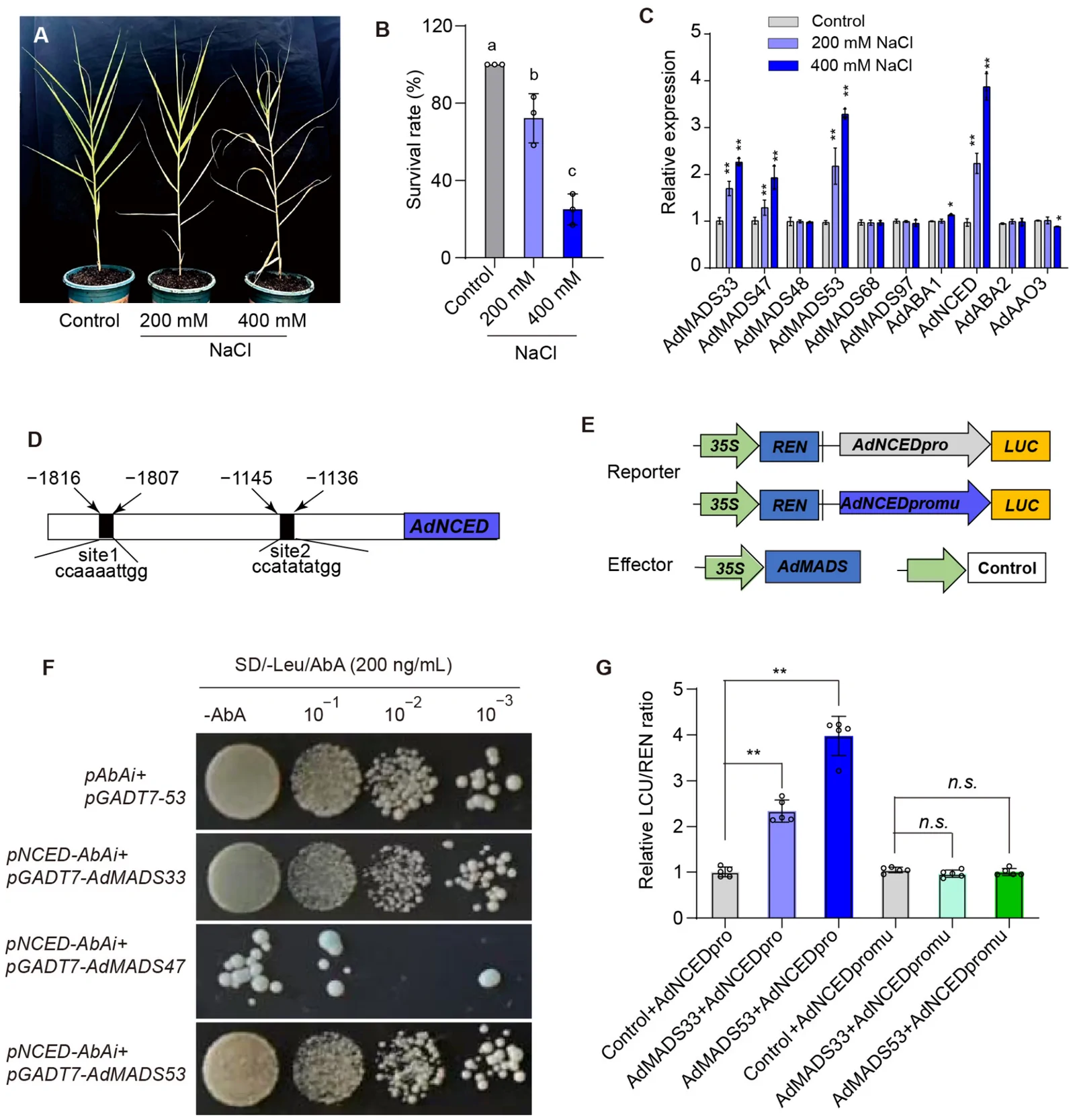
AdMADS33 and AdMADS53 promote transcription of *AdNCED*. (**A**) Morphology of the *A. donax* seedling under normal, 200 mM NaCl, or 400 mM NaCl treatment. Scale bar = 10 cm. (**B**) Survival rate of *A. donax* seedlings under normal or 200/400 mL NaCl conditions. Data are presented as mean ± SD (*n* = 3 biological replicates). Different letters indicate significant differences at *p < 0.05* based on one-way ANOVA and Tukey’s HSD test. (**C**) Expression levels of six selected *AdMADS* genes and four ABA synthesis genes under salt (200/400 mM NaCl) treatments, as determined by RT-qPCR. Data are presented as mean ± SD (*n* = 3 biological replicates). (**D**) Schematic diagram of the two CArG motifs in the *AdNCED* promoter region. (**E**) Yeast one-hybrid assays were performed by co-transforming yeast Y1HGold cells, followed by screening on selective medium. pGADT7-53 + pAbAi served as the positive control, while pAdNCED-AbAi + pGADT7-AdMADS served as the experimental groups. Aureobasidin A (AbAr) was used at 200 ng/mL. (**F**) Schematic diagram of vector construction for dual luciferase assays. *AdNCED* promoter (*AdNCEDpro*) or *AdNCED* promoter without CArG motif (*AdNCEDpromu*) driving the LUC genes was used as reporter, and the 35S promoter driving *AdMADS* genes was used as effector. (**G**) Relative LUC/REN luciferase activity of different groups. Data are presented as mean ± SD (*n* = 5 biological replicates). Asterisks indicate a significant difference as determined by Student’s *t*-test (** *p* < 0.01, * *p* < 0.05, *n.s.*, no significant difference).

ABA is one of the most important hormones affecting abiotic stress tolerance in plants [27]. Previous studies have identified several MADS-box genes that regulate salt stress tolerance through ABA signaling, such as OsMADS23 [17] and OsMADS25 [18]. Among the *AdMADS* genes in *A. donax*, six were up-regulated by salt stress (Figure 6C). To determine whether these MADS-box genes affect ABA synthesis in *A. donax*, we treated *A. donax* seedlings with 200 mM NaCl and 400 mM NaCl (Figure 8A). The seedlings showed a much higher survival rate (72.2%) under 200 mM NaCl than under 400 mM NaCl (25.0%) (Figure 8B). We then quantified the expression levels of the *AdMADS* genes and ABA synthesis genes. Among the six *AdMADS* genes, *AdMADS33*, *AdMADS47*, and *AdMADS53* were up-regulated, whereas the expression of the other three *AdMADS* genes showed no obvious change. Among the four ABA synthesis genes (*AdABA1*, *AdNCED*, *AdABA2*, and *AdAAO3*), *AdNCED* was significantly up-regulated by salt stress, both under 200 mM NaCl and 400 mM NaCl treatment, while there was a slight change or no obvious change in the other three genes (Figure 8C). Therefore, we speculated that AdMADS may regulate the transcription of *AdNCED*. Interestingly, two CArG boxes were found in the *AdNCED* promoter region (Figure 8D). We used a yeast one-hybrid system (Y1H) to test whether AdMADS33, AdMADS47, and AdMADS53 can directly bind to the *AdNCED* promoter, and the results showed that AdMADS33 and AdMADS53 can bind to the CArG box in the *AdNCED* promoter region (Figure 8E). To further examine the transcriptional regulation of *AdNCED* by AdMADS33 and AdMADS53, we performed transient transcription assays in *Nicotiana benthamiana* (*N. benthamiana*) leaves using the dual-luciferase (LUC) reporter system (Figure 8F). Coexpression of *AdMADS33* or *AdMADS53* significantly enhanced the LUC activity driven by the *AdNCED* promoter, whereas this transcriptional activation was abolished when the two CArG motifs in the *AdNCED* promoter were mutated, confirming the transcriptional activation of AdMADS33 and AdMADS53 to *AdNCED* (Figure 8G). These results demonstrate that AdMADS33 and AdMADS53 can bind to and activate the *AdNCED* promoter in heterologous systems. However, these data do not establish in planta regulation of *AdNCED* by AdMADS33/AdMADS53, and further genetic evidence is required to determine whether they positively regulate salt tolerance in *A. donax*.

## 3. Discussion

### 3.1. MADS-Box Genes in A. donax Showed a Unique Distribution in Subgroups

MADS-box genes are key regulators of plant growth, development, and abiotic stress responses. The functions of MADS-box genes have been investigated in a number of species, including the model plant Arabidopsis [28], as well as key cereals such as rice [29], wheat [30], and barley [31], among others. In this study, we provided the first genome-wide characterization of the MADS-box gene family in *A. donax* and identified 45 stress-responsive AdMADS candidates. We further show that AdMADS33 and AdMADS53 bind to the *AdNCED* promoter and activate its transcription in a heterologous reporter system, suggesting a link between MADS-box TFs and ABA-mediated salt tolerance. These findings offer clues for future functional studies and for identification of candidate genes for molecular breeding. However, in planta gain- and loss-of-function experiments are needed to establish the physiological functions of these genes before they can be used as breeding targets.

Our analysis revealed significant differences in the physicochemical properties of the encoded proteins and gene structures between type I and type II *AdMADS* genes. Consistent with findings in species like Arabidopsis and rice [28,29], we observed a striking divergence in gene structure between the two types: type II *AdMADS* genes possess significantly more introns than type I *AdMADS* genes. This pattern suggests distinct evolutionary trajectories, potentially involving extensive intron loss in type I genes, which may have conferred a selective advantage during the evolution of *A. donax*.

Phylogenetic comparison with other gramineous plants such as rice and foxtail millet revealed a conserved distribution of type I MADS genes in *A. donax* (Figure 2). In contrast, the type II subfamily exhibited a distinct pattern, most notably through the absence of the AGL6 and MIKC* subgroups. Notably, these two subgroups are known to be critical for flower development: AGL6 genes regulate floral meristem maintenance, organ identity, and ovule development [32], while MIKC* genes are essential for pollen maturation and tube growth [33]. *A. donax* is characterized by sterility and aberrations in gametogenesis [34]. The absence of these specific MADS-box subgroups, which are fundamental to reproductive development, could be a contributing factor to this sterile phenotype. However, sterility in *A. donax* is a complex, multifactorial phenomenon that cannot be attributed to MADS-box gene loss alone. In fact, *A. donax* sterility has been reported to be associated with alterations in both gametogenesis and fertilization, as well as post-fertilization [34]. Therefore, future research aimed at restoring fertility in *A. donax* could investigate the potential of genetic engineering approaches to reintroduce functional orthologs of the specific MADS-box genes, while also considering the broader developmental and physiological factors that contribute to sterility.

### 3.2. AdMADS Genes Play Essential Roles in Abiotic Stress Resistance

In this study, a greater number of *AdMADS* genes responded to waterlogging stress (35 genes) compared to drought (6 genes) and salt stress (17 genes) (Figure 6 and Figure S1C), suggesting a particularly prominent role for this gene family in *A. donax*’s adaptation to waterlogging. Among the 35 waterlogging-responsive *AdMADS* genes, the majority (25 genes, 71.4%) were differentially expressed in both leaf and root tissues. A subset of genes exhibited consistent regulation across tissues; for example, *AdMADA4*, *AdMADS25*, *AdMADS50*, and *AdMADS54* were up-regulated, while *AdMADS18*, *AdMADS29*, and *AdMADS64* were down-regulated in both leaves and roots. In contrast, other genes displayed tissue-specific expression patterns, such as *AdMADS26* (up-regulated in leaf but down-regulated in root) and *AdMADS62* (down-regulated in leaf but up-regulated in root) (Figure 6A). These results highlight the complexity of the transcriptional network underlying *A. donax*’s response to waterlogging stress. Waterlogging primarily causes hypoxia and altered energy metabolism [35]. MADS-box genes may participate in these responses through crosstalk with ABA, ethylene, ROS, and hypoxia-related pathways. Future studies could examine the relationship between *AdMADS* genes and known hypoxia-responsive pathways such as N-degron oxygen-sensing [36,37].

Supporting the established roles in abiotic stress responses of MADS-box genes [38,39], our study identified 45 *AdMADS* genes that were differentially expressed under waterlogging, drought, and salt stresses. A notable finding was the significant overrepresentation of type II genes among these stress-responsive *AdMADS* members, accounting for 91.1% (41 out of 45) (Table S8). This predominance of type II genes aligns with observations in other species; for instance, in barley, 69.6% of abiotic stress-responsive *HvMADS* genes were also from the type II subfamily [33]. This trend is further corroborated by functionally characterized type II genes, such as *AGL16* in Arabidopsis, and *OsMADS23*, *OsMADS25*, *OsMADS27* in rice [16,17,18], and *SiMADS51* [40] in foxtail millet, which have been demonstrated to mediate adaptation to various abiotic stresses. Collectively, these findings underscore that MADS-box genes, especially type II genes, are major contributors to the transcriptional reprogramming underlying plant abiotic stress tolerance. However, it should be noted that differential expression alone does not demonstrate a functional role in stress tolerance; genetic validation is required.

### 3.3. AdMADS33 and AdMADS53 Are Candidate Regulators of AdNCED Transcription

Among the salt-responsive *AdMADS* genes, *AdMADS33*, *AdMADS47* and *AdMADS53* were selected for further analysis because they were significantly up-regulated under salt stress. Y1H and dual-LUC assays demonstrated that AdMADS33 and AdMADS53 can bind to the ABA synthesis gene *AdNCED* promoter and activate its transcription in heterologous systems. These results suggest a possible regulatory module in which AdMADS33/AdMADS53 promote ABA synthesis by activating *AdNCED*, thereby potentially contributing to salt tolerance. However, it is important to note that the current evidence is based on heterologous reporter systems and does not establish in planta regulation of *AdNCED* or a salt-tolerance phenotype. Direct genetic evidence from overexpression, knockout, or silencing lines is required to confirm the physiological functions of AdMADS33 and AdMADS53 in *A. donax*.

Notably, among the four ABA biosynthesis genes examined, only *AdNCED* was significantly up-regulated under both 200 mM and 400 mM NaCl treatments, whereas *AdABA1*, *AdABA2*, and *AdAAO3* showed no obvious changes (Figure 8B). This pattern may reflect the fact that NCED catalyzes a rate-limiting step in ABA biosynthesis, and its transcriptional up-regulation alone may be sufficient to increase ABA production under salt stress [27]. In contrast, ABA1, ABA2, and AAO3 may be regulated at other levels, such as post-transcriptional or post-translational modification, or may respond at different time points or in different tissues not captured in our experimental conditions. In addition, feedback regulation of the ABA pathway could buffer the expression of these upstream or downstream genes. Therefore, the salt-induced up-regulation of *AdNCED*, together with the binding and activation of its promoter by AdMADS33 and AdMADS53, suggests a possible AdMADS-*AdNCED*-ABA module in salt response. However, this hypothesis remains to be tested by direct measurements of ABA content and in planta genetic experiments.

## 4. Materials and Methods

### 4.1. Identification of AdMADS Genes in A. donax Genome

Genome assembly, genome annotation, and protein sequences of *A. donax* were obtained from the China National Center for Bioinformation (https://www.cncb.ac.cn/) [21]. To identify all putative MADS-box genes in *A. donax*, we first retrieved the protein sequences of 76 known MADS-box genes from the rice genome (URL: https://rice.uga.edu/cgi-bin/putative_function_search.pl. Accessed on 2 August 2025) and used them as a query to perform a BLASTP search against the *A. donax* proteome. Subsequently, the hidden Markov models (HMMs) for the SRF-TF (PF00319) and K-box (PF01486) domains were obtained from the Pfam database (URL: https://www.ebi.ac.uk/interpro/entry/pfam/. Accessed on 2 August 2025). These HMM profiles were then used to screen the initial candidate set with the Pfam-search tool, retaining only sequences with an e-value <0.05. The number of amino acids, molecular weight, isoelectric point, and instability index for each identified protein were predicted using the ExPASy ProtParam online tool (URL: https://web.expasy.org/protparam/. Accessed on 4 August 2025) [41]. The chromosomal locations of the *AdMADS* genes were determined directly from the *A. donax* genome annotation file and visualized with TBtools software v2.322 [42].

### 4.2. Phylogenetic Analysis of AdMADSs

Multiple sequence alignment of the identified MADS-box proteins from *A. donax*, rice, and Arabidopsis was performed using the MUSCLE software v5.1 [43]. The phylogenetic tree based on the maximum-likelihood method was constructed from the alignment using IQ-TREE v 2.4.0 [44], with branch support assessed by 1000 bootstrap replicates. The final tree was visualized and annotated using the R package ggtree v3.16.0 [45].

### 4.3. Gene Structure, Conserved Domain and Motif, and Cis-Acting Elements Analysis

Conserved protein domains were identified using the online CD-Search tool [46] (URL: https://www.ncbi.nlm.nih.gov/Structure/cdd/wrpsb.cgi. Accessed on 6 August 2025). Conserved motifs were predicted with the MEME suite [47] (ULR: https://meme-suite.org/meme/tools/meme. Accessed on 6 August 2025), with the maximum number of motifs set to 20 and all other parameters at their default values. The 2000-bp promoter sequences upstream of the start codon (ATG) of each *AdMADS* gene were analyzed for prediction of cis-regulatory elements using the PlantCARE database [48] (URL: http://bioinformatics.psb.ugent.be/webtools/plantcare/html/. Accessed on 7 August 2025). Gene structures, conserved motifs, protein domains, and the distribution of cis-regulatory elements were visualized using the TBtools II software v2.322 [29].

### 4.4. Gene Duplication and Collinearity Analysis

Putative duplicated gene pairs within the *A. donax* genome were identified using MCScanX v1.1 [49] software with default parameters. The resulting gene pairs were displayed using TBtools II v2.322, with the *AdMADS* genes highlighted. To investigate their evolutionary trajectories and test for positive selection, the Ka (nonsynonymous) and Ks (synonymous) substitution rates, along with the Ka/Ks ratios for the duplicated *AdMADS* gene pairs, were calculated using KaKs_Calculator 3.0 [50]. The divergence time (T) for each gene pair was estimated using the formula T = Ks/2λ, where λ (here equals 6.5 × 10^−9^) represents the commonly used divergence rate for nuclear genes in monocotyledonous plants [51]. Collinearity relationships of the MADS-box gene families among *A. donax*, rice, and foxtail millet (*Setaria italica*) were analyzed using JCVI software v1.5.6. The syntenic plots were generated using the command python -m jcvi.graphics.karyotype.

### 4.5. RNA-Seq Analysis

The RNA-seq datasets of *A. donax* under various stress conditions were downloaded from the NCBI SRA database [24,25,26]. The accession numbers, plant tissues, developmental stages, stress duration, stress intensity, biological replicates, and sequencing platforms were listed in Table S7. The raw reads were first processed with fastp v0.23.2 [52] to remove low-quality bases and adapter sequences. The clean reads were then mapped to the *A. donax* reference genome using HISAT2 v2.2.1 [53], and the resulting alignments were sorted into BAM files using SAMtools v1.6 [54]. Read counts for each gene were quantified using featureCounts v2.0.8 [55], followed by normalization to transcripts per million (TPM) and visualization as a heatmap using R. Differentially expressed genes (DEGs) were identified using the DESeq2 package [56] with false discovery rate (FDR) <0.05 and log_2_(fold change) >1.

### 4.6. Plant Material and Treatment

The plant material used in this study originated from a single *A. donax* individual collected in Wuhan, Hubei, China (114.276° E, 30.789° N). Seedlings were propagated and maintained in vitro on MS medium [57] and subsequently acclimatized and grown in a nursery during the spring of 2024. For the stress treatments, uniform seedlings at the four-leaf stage were transplanted into pots (30 cm in diameter and height). All plants were grown in a controlled greenhouse under the following conditions: a 28/22 °C day/night temperature regime, 60% relative humidity, and a 16-h photoperiod. The abiotic stress treatments were maintained for four weeks. Waterlogging stress was applied by flooding the pots to maintain the water level at half the height of the aboveground plant parts. Drought and salt stresses were imposed by irrigating the plants with a nutrient solution containing 20% (*w*/*v*) PEG-6000 or 200/400 mmol/L NaCl, respectively. Control plants were irrigated with the standard nutrient solution throughout the experiment.

### 4.7. Real-Time qPCR Assays

Total RNA was extracted from leaf, shoot, and root tissues using a plant RNA isolation kit (TIANGEN, D9419). Reverse transcription-quantitative PCR (RT-qPCR) was conducted using a one-step SYBR Green kit (Yeasen, 11213ES, Shanghai, China) on an Applied Biosystems 7500 Real-Time PCR System. The relative expression levels were calculated using the 2^−ΔΔCt^ method [58], with the *A. donax* actin gene serving as the internal reference. The sequences of all RT-qPCR primers are listed in Table S1.

### 4.8. Yeast One-Hybrid Assays

The promoter fragment (2000 bp upstream of the start codon) of *AdNCED* was amplified by PCR and cloned into the *Hind*III and *Kpn*I sites of the *pAbAi* reporter vector to generate the bait construct *pAdNCED-AbAi*. Simultaneously, the coding sequence of *AdMADS33*, *AdMADS47,* or *AdMADS53* was cloned into the *EcoR*I and *Sac*I sites of the *pGADT7* prey vector to generate the prey construct *pGADT7-AdMADS33*, *pGADT7-AdMADS47*, or *pGADT7-AdMADS53*. The recombinant bait vector was transformed into the yeast strain Y1HGold, and the transformed cells were plated on SD/-His/-Leu dropout medium containing various concentrations of Aureobasidin A (AbA) to determine the minimum inhibitory concentration required to suppress background auto-activation. Subsequently, *pAdNCED-AbAi* and *pGADT7-AdMADS* were co-transformed into Y1HGold yeast competent cells, and the transformation products were plated on SD/-His/-Leu selective medium supplemented with the optimal AbA, followed by incubation at 30 °C for 2–4 days. Primers were listed in Supplementary Table S1.

### 4.9. Dual-Luciferase Assay

The reporter vector was constructed by inserting the ~2000 bp *AdNCED* promoter upstream of the LUC reporter gene in the *pGreenII 0800-LUC* vector. As a specificity control, we generated a mutant reporter carrying two destroyed CArG-box motifs within the same promoter using a Q5 site-directed mutagenesis kit (NEB). The effector vector *pGreenII 62-SK* was used to express *AdMADS33 and AdMADS53* under a strong constitutive promoter. Each reporter construct was co-transformed with either the effector or an empty vector control into Agrobacterium GV3101, and the cultures were infiltrated into *N. benthamiana* leaves. Following a 60 h incubation, leaf disks were harvested for dual-luciferase activity measurement (Promega E1910), with Renilla luciferase serving as an internal normalization control. Primer sequences are detailed in Supplemental Table S1.

## 5. Conclusions

As a promising bioenergy crop, *A. donax* requires in-depth molecular characterization to support its genetic improvement. This study presents the first comprehensive genome-wide identification and characterization of the MADS-box gene family in *A. donax*. We systematically analyzed their phylogenetic relationships, chromosomal distributions, gene structures, conserved protein motifs and domains, and collinearity relationships. Furthermore, by integrating RNA-seq and RT-qPCR analyses, we identified 45 *AdMADS* genes that respond to waterlogging, drought, and salt stresses. Among them, AdMADS33 and AdMADS53 were shown to bind to and activate the *AdNCED* promoter in heterologous systems, suggesting that they may contribute to salt tolerance by promoting *AdNCED* transcription. However, direct in planta genetic validation is required to confirm their physiological functions. Our findings establish a foundation for future functional studies and provide valuable genetic resources for the molecular breeding of stress-resilient *A. donax* varieties. Future work should focus on the functional characterization of key candidate genes, elucidation of their regulatory networks, and validation of their roles through transgenic assays.

## Figures and Tables

**Table 1:** Comparison of the physicochemical properties of two types of MADS-box proteins.

Type of MADS-Box Proteins	Amino Acids Numbers	Molecular Weight (KDa)	Isoelectric Point	Instability Index	Hydropathicity (GRAVY)
Type I (mean ± SD)	300.93 ± 102.46	32.68 ± 11.12	7.00 ± 1.72	52.14 ± 10.27	−0.401 ± 0.244
Type II (mean ± SD)	238.43 ± 66.57	27.30 ± 6.99	7.98 ± 1.38	54.55 ± 7.28	−0.630 ± 0.181
*p* value of Student’s *t*-test	0.0009	0.007	0.003	0.195	2.0 × 10^−6^

## Data Availability

The data presented in this study are available within the article and Supplementary Materials.

## Supplementary Materials

The following supporting information can be downloaded at: https://www.mdpi.com/article/10.3390/plants15213267/s1, Figure S1: Statistics of DEG *AdMADS* genes; Table S1: Primers used in this study; Table S2: The predicted feature of 97 AdMADSs; Table S3: Statistics of gene stuctures of *AdMADS* genes; Table S4: Cis-regulatory elements in *AdMADS* promoters; Table S5: Collinearity relationship of AdMADS genes; Table S6: Collinearity of the MADS-box genes among species; Table S7: Information of RNA-seq datasets used in this study; Table S8: Differentially expressed *AdMADS* genes.
