# Supplementary material for: Genome-Wide Characterization and Expression Profiling of MADS-Box Family Genes Under Abiotic Stress in Arundo donax
Source: Plants (Basel). 2026 Oct 23;15(21):3267. doi: 10.3390/plants15213267 (PMC13765611; doi:10.3390/plants15213267)
Supplement: Supplementary file 1 [file plants-15-03267-s001.zip › Figure S1. Statistics of DEG AdMADS genes.pdf]

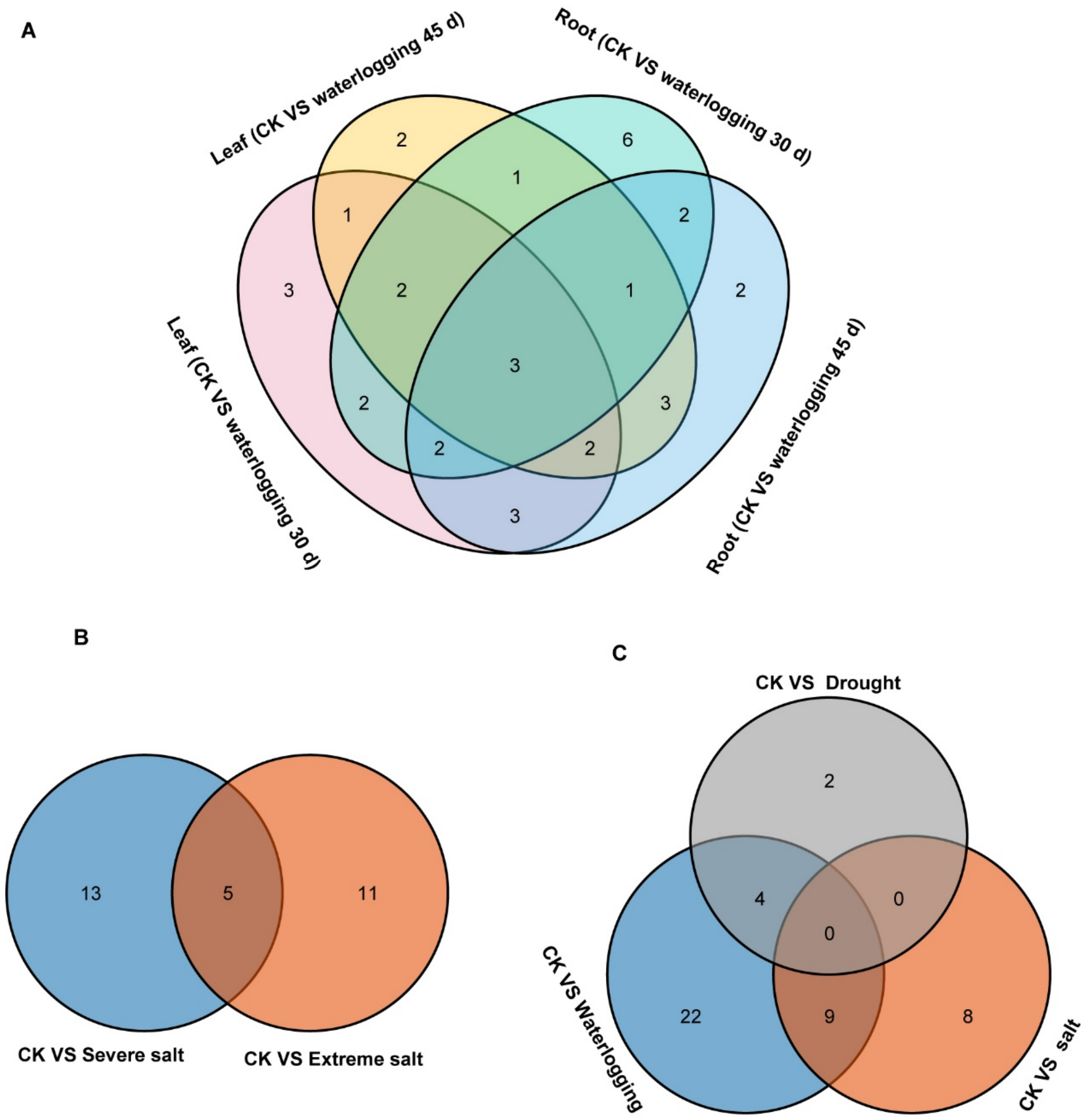

Figure S1. Statistics of differentially expressed *AdMADS* genes response to abiotic stress. Veen diagrams of differentially expressed genes under different waterlogging stress in root and shoot tissues (A), different salt stress conditions (B). (C) Comparison of differentially expressed genes under three stress conditions.
